# Seven-month wintertime supplementation of 1200 IU vitamin D has no effect on hand grip strength in young, physically active males: A randomized, controlled study

**DOI:** 10.1080/15502783.2022.2100718

**Published:** 2022-07-19

**Authors:** Leho Rips, Alar Toom, Rein Kuik, Ahti Varblane, Hanno Mölder, Marika Tammaru, Mart Kull, Vahur Ööpik, Jüri-Toomas Kartus, Helena Gapeyeva, Madis Rahu

**Affiliations:** a Tartu University Hospital, Sports Medicine and Rehabilitation Clinic, Tartu, Estonia; b University of Tartu, Department of Sports Medicine and Rehabilitation, Institute of Clinical Medicine, Faculty of Medicine, Tartu, Estonia; c Central Finland Central Hospital, Department of Orthopaedics, Jyväskylä, Finland; d Estonian National Defence College, Centre of Military Disaster Medicine, Tartu, Estonia; e Medical Centre of the 2nd Infantry Brigade CSS Battalion, Estonian Defence Forces, Võru, Estonia; f East-Tallinn Central Hospital, Research Department, Tallinn, Estonia; g Viljandi Hospital, Jämejala Viljandi, Estonia; h University of Tartu, Institute of Sport Sciences and Physiotherapy, Tartu, Estonia; i University of Gothenburg, Institute of Clinical Siences, Sahlgrenska Academy, Göteborg, Sweden; j East-Tallinn Central Hospital, Clinic of Medical Rehabilitation, Tallinn, Estonia

**Keywords:** Military training, hand grip, vitamin D, supplementation

## Abstract

**Background:**

There has been a growing interest in the role of vitamin D for the well-being and physical performance of humans under heavy training such as conscripts in military service; however, there is a lack of long-term supplementation studies performed on members of this type of young, physically active, male population. The hypothesis of the study was that vitamin D supplementation during wintertime will decrease the prevalence of critically low vitamin D blood serum levels and increase hand grip strength during the winter season among young male conscripts.

**Study Design:**

Longitudinal, triple-blinded, randomized, placebo-controlled trial.

**Methods:**

Fifty-three male conscripts from the Estonian Army were randomized into two groups: 27 to an intervention group and 26 to a placebo group. The groups were comparable in terms of their demographics. The intervention group received 1200 IU (30 µg) capsules of vitamin D3, and the control group received placebo oil capsules once per day. The length of the follow-up was 7 months, from October 2016 until April 2017. Blood serum vitamin D (25(OH)D), parathyroid hormone (PTH), calcium (Ca), ionized calcium (Ca-i), testosterone and cortisol values, and hand grip strength were measured four times during the study period.

**Results:**

The mean 25(OH)D level decreased significantly in the control group to a critically low level during the study, with the lowest mean value of 22 nmol/l found in March 2017. At that time point, 65% in the control group vs 15% in the intervention group had 25(OH)D values of less than 25 nmol/l (p < 0.001). In the intervention group, the levels of 25(OH)D did not change significantly during the study period. All other blood tests revealed no significant differences at any time point. The corresponding result was found for hand grip strength at all time points.

**Conclusion:**

Long-term vitamin D supplementation during wintertime results in fewer conscripts in the Estonian Army with critically low serum vitamin D (25(OH)D) levels during the winter season. However, this did not influence their physical performance in the form of the hand grip strength test.

## 1. Introduction

Interest in the role of vitamin D in humans has been growing. Despite the large number of papers published during the last few decades, there is still a lack of knowledge in terms of seasonal variation and need for vitamin D supplementation in persons with a physically demanding lifestyle; this includes differences between age groups and gender subpopulations in both healthy persons and patients. The key role of vitamin D in musculoskeletal health is well known, especially the morbidity of its depletion such as abnormal bone metabolism and risk of osteopenia and osteoporosis, muscle weakness, and increased risk in fractures [1–4]. However, it is also known that nonmusculoskeletal disorders such as diabetes, metabolic disorders, infectious diseases caused by decreased immunity, autoimmune diseases, and hypertension are, potentially, adversely affected by a lack of vitamin D [5]. The effect of vitamin D on physical performance is still controversial, and there is a lack of evidence in terms of its effect on hand grip strength [6,7].

Vitamin D is a unique form of hormone and has receptors in most cells and tissues. It has a wide range of biological actions, including key roles in calcium metabolism and bone remodeling [8] as well as at receptor sites in muscle tissue, potentially having a crucial role in muscle function [9]. It is also known that vitamin D can affect the metabolism of testosterone and cortisol in the human body [10–12], both of which are known to be important in physical performance and level of stress [13].

Uncovered skin contact with UV radiation is the main mechanism of vitamin D synthesis in the human body [14]. Certain forms of vitamin D, such as D2 (found in mushrooms) and D3 (fish, eggs, and meat), are the main natural food sources for humans [15]. Supplements and fortified foods are additional means of obtaining adequate amounts of vitamin D to meet dietary needs. Vitamin D is inactive in the human body and needs a two-step hydroxylation: first in the liver to form 25(OH)D and second in the kidneys to produce the biologically active form 1,25(OH)2D [16]. The main form of vitamin D in blood serum is 25(OH)D, and this is used as an indicator of vitamin D status [17]. In a study by Funderburk et al. [18], subjects in a military environment were classified by their vitamin D status. In this study, vitamin D was considered sufficient if the serum 25(OH)D concentration was ≥ 75 nmol/l, insufficient if between 51 and 75 nmol/l, and deficient if < 50 nmol/l. Based on an Estonian population study by Kull et al. in 2009 [19], critical deficiency was determined as a serum concentration of < 25 nmol/l. Furthermore, deficiency has been determined as < 50 nmol/l and insufficiency as < 75 nmol/l by the Endocrine Society (ES) [1]. There is no consensus concerning the normal level of serum 25(OH)D. Some studies have even defined the insufficiency boundary as high as 80 nmol/l [20,21]. Personal characteristics, such as skin pigmentation, age, type of clothing, use of sunscreen, physical and outdoor activity, and sun exposure, can prevent or promote vitamin D synthesis and will influence serum 25(OH)D levels [22]. A low level of vitamin D is a well-known problem in northern European countries [19,23]. However, in recent studies, an increasing tendency toward this has also been reported in the southern part of Europe and at higher northern latitudes (above 40°N; e.g. Madrid). Sun exposure during winter activates only low levels of dermal generation of vitamin D. Thus, there is an increased need in supplementation [23,24].

A population-based study performed in Estonia found the mean serum 25(OH)D concentration to be 43.7 nmol/l during winter and 59.3 nmol/l during summer. The authors of that study stated that vitamin D deficiency is highly prevalent throughout the year in this population, who live at a latitude of 59°N without vitamin D dairy product supplementation [19]. A large Finnish-population study also found vitamin D deficiency in all age groups during the winter season [25], and there are similar seasonal variations reported in other European studies [1,2,5,23–29]. A similar finding of widespread vitamin D deficiency among soldiers was presented in a recent prospective cohort study performed in the US Army [30].

Routine dietary fortification of vitamin D is common practice in Norway, Sweden, and Finland. These countries therefore have better basic levels of serum 25(OH)D among their populations than Estonia [25,28–31]. There is no official dairy fortification with vitamin D in Estonia, which increases the risk of having a low level of serum 25(OH)D [19].

Muscle fitness is crucial in military service. To measure physical fitness, the hand grip strength test is easy to perform and it is widely used to measure upper body muscle fitness in all age groups [32–37] and is often used as an indicator of general health. Carswell et al. [4] found evidence that low 25(OH)D levels were negatively related to muscle fitness in the UK army. Other studies show that lower muscle fitness and hand grip strength are related to higher risk of cardiovascular disease events and mortality in middle-aged individuals [38] and an increased suicide risk in adolescents [39]. Another recent prospective cohort study in the UK Army reported that vitamin D is clearly associated with endurance performance; however, it showed that power and strength are not affected by vitamin D [4]. There is evidence that vitamin D supplementation has a positive effect on upper limb muscle strength [6]. There is lack of knowledge on how vitamin D deficiency and supplementation affect hand grip strength in young, healthy individuals, such as conscripts.

Therefore, a prospective, randomized, blinded study in young male conscripts in the Estonian army was designed. The hypothesis of the study was that without oral vitamin D supplementation, vitamin D deficiency occurs during the winter season and that this can cause a decrease in hand grip strength.

The primary outcome of the study was the serum levels of 25(OH)D during the study period. The secondary outcome of the study was the hand grip strength.

## 2. Materials

All conscripts (n = 403) entering military service in October 2016 at the Kuperjanov Battalion, Võru, Estonia (situated at a latitude of 58°N, which corresponds to that of southern Alaska in the US and Quebec in Canada), were asked to participate in the first briefing of this study. The recruits were informed of the purpose of the study, recruitment criteria, and follow-up methods. Only those who volunteered to participate in the study and signed informed consent after this first briefing were included in the study. A total of 65 conscripts volunteered to participate initially, and 63 of these returned their informed consent.

Ten conscripts, five in the intervention group and five in the control group; were later excluded from the study due to premature cessation of their military service: two for mental health problems, two for lower-back pain, one for polyarthritis, and five for other medical reasons. Data from 53 conscripts, all of Caucasian origin, were included in the final analysis: 27 in the intervention group and 26 in the control group. The anthropometric characteristics of the study groups are presented in Table 1. The only exclusion criterion was the inability to continue military service, for any reason, during the 7-month follow-up period.

**Table 1.** Anthropometric characteristics at the baseline and during follow-up of study groups.

	Intervention group	Placebo group	Significance
Number of participants	27	26	
Age (years) Median (range) Mean (SD)	21 (19–27)20.8 (1.7)	21 (19–26)21.2 (2.0)	n.s. (0.38)
Height (cm) Median (range) Mean (SD)	180 (167–191)180 (6.9)	181 (163–191)179.0 (7.7)	n.s. (0.49)
Weight baseline (October 2016) (kg) Median (range) Mean (SD)	72.0 (55.7–97.7)74.0 (10.8)	75.6 (49.3–95.2)74.5 (11.1)	n.s. (0.89)
BMI baseline (October 2016) kg/m^2^ Median (range) Mean (SD)	22.0 (19.5–28.2)22.7 (2.4)	23.1 (18.1–26.1) 23.2 (2.6)	n.s. (0.48)
Weight follow-up III (April 2017) (kg) Median (range) Mean (SD)	75.0 (61.4–100.2)76.8 (10.3)	77.2 (56.9–96.0)77.4 (10.6)	n.s. (0.85)
BMI follow-up III (April 2017) kg/m^2^ Median (range) Mean (SD)	23.3 (19.6–28.9)23.5 (2.1)	24.1 (20.4–28.1)24.1 (2.4)	n.s. (0.37)

Abbreviations: SD, standard deviation; BMI, body mass index; n.s., nonsignificant

The weight and BMI increased significantly between baseline and follow-up III in both groups (p < 0.001).

**Figure 1.**
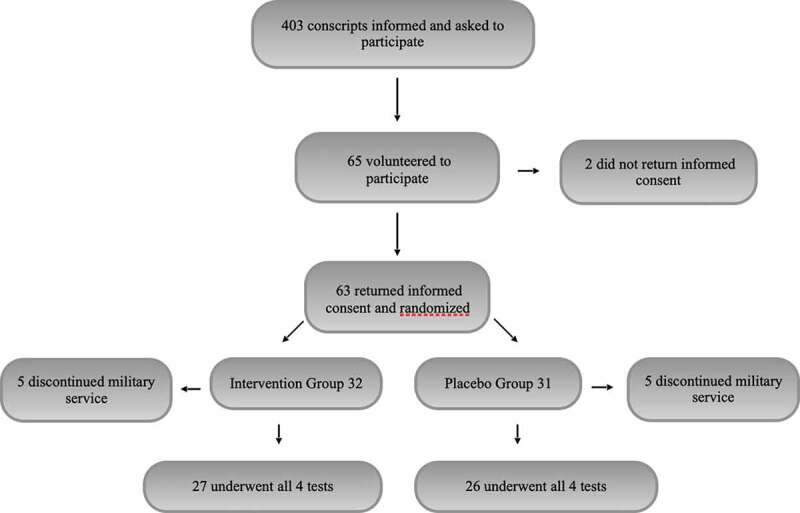
Flowchart of the study.

A flowchart of the study is presented in Figure 1.

In both study groups, the conscripts followed routine military preparations based on the guidelines of the Estonian Defense Forces. Ordinary daily routines start at 6 AM and end at 10 PM, with rest at night. There are three main meals served and two additional snacks provided between lunch and dinner. Military training starts with 10 weeks of basic training consisting of physical preparations and special training for different military needs. Physical training is focused on increasing aerobic and anaerobic capacity, varying from the minimum of 1–2 hours of daily training to a 50 km extreme hike in a complex landscape with full military equipment. The basic training program consists of a daily morning run up to 5 km, strength training using the body weight, and maximum muscle strength training in the gym. The following 4.5 months consist of special skills training, physical performance training, self-defense training, urban and forest combat training, and special tactical and weapon training.

Upper body strength is important in combat situations, handling weapons and to carry heavy weight, and thus, the hand grip strength is a relevant test to measure physical fitness.

The study was approved by the Research Ethics Committee of the University of Tartu No. 262/T-28 and 264 M-14 and funded by grant No. R-002 of Estonian Defense Forces.

## 3. Study design and data collection

A longitudinal, triple-blinded (participants, supervisors, and researchers), randomized, placebo-controlled trial (ClinicalTrials.gov NCT04359524) with a 7-month follow-up period from October 2016 until April 2017 was performed.

The body mass (kg) and height (cm) of the conscripts were measured two times by the same nurse at the Kuperjanov Battalion medical center using standardized equipment, and their body mass index (BMI) was calculated in kg/m^2^.

Computed randomization was used to divide conscripts into two groups: either the intervention group, in which conscripts received vitamin D3 capsules (1200 IU/30 µg), or the control group, in which conscripts received a placebo (olive oil capsules). Both types of capsules were standardized for size and color, administered once per day, in the morning before breakfast, for 7 months. Every conscript was provided a personal copy of the information with explanations on the supplementation. The general and daily supplementation protocols were supervised by a member of the local medical staff who had undergone special training.

Standardized coded packages (three per conscript) and capsules (100 per package) were manufactured on special order by Innopharma A/S (Denmark). No commercial sponsoring was involved. The key to the package code numbers was stored in a computer database until the unblinding of the participants. At the end of the study. all the packages and capsules were collected and destroyed.

A dosage of 1200 IU of vitamin D3 per day was used, because this was the maximum recommended daily supplementation dosage stated by the Estonian State Agency of Medicines (Personal communication, based on regulation No. 59 of the Minister of Social Affairs of the Republic of Estonia, April 13^th^, 2005) at the time of the study.

The hand grip test was performed using a validated hydraulic hand dynamometer (Lafayette Instrument Co., USA). Measurements were taken from each participant in the standing position, arms at the side, not touching the body, with the elbow slightly bent. The participant squeezed the dynamometer with as much force as possible. The best result of three trials, with pause of about 10–20 seconds between the trials, was recorded in kilograms. The same procedure was performed for both hands.

Blood serum values of vitamin D (25(OH)D), parathyroid hormone (PTH, normal 1.48–7.83 pmol/l), calcium (Ca, normal 2.15–2.6 mmol/l), ionized calcium (Ca-i, normal 1.12–1.32 mmol/l), testosterone (normal 8.4–28.7 nmol/l), and cortisol (normal 138–690 nmol/l) were measured four times during the study period: first in October 2016 to provide baseline values and then subsequently in December 2016, March 2017, and April 2017. All blood samples were subjected to overnight fasting tests, collected on the same day of the week, all within the same hour, and under standardized conditions.

## 4. Laboratory measurements

Serum samples for clinical chemistry analysis were collected in serum clot activator tubes (BD Vacutainer SST II Advance Plus Blood Collection Tubes, Becton Dickinson and Company, New Jersey, United States). Calcium measurements were performed using the spectrophotometry method (ADVIA® 1800 Clinical Chemistry System, Siemens Healthcare GmbH, Erlangen, Germany). Ionized calcium measurements were performed using ion selective electrodes (AVL 9180 Electrolyte Analyzer, Roche Diagnostics, Germany). The direct chemiluminescence immunoassay method was used for the measurement of PTH (ADVIA Centaur XP, Siemens Healthcare GmbH, Erlangen, Germany). Measurements of 25(OH)D were performed using the direct chemiluminescence immunoassay method (LIAISON XL, DiaSorin S.p.A, Saluggia VC, Italy). The direct chemiluminescence immunoassay method was used for the measurement of testosterone (ADVIA Centaur TSTII assay, Siemens Healthcare GmbH, Erlangen, Germany). The solid-phase competitive chemiluminescence enzyme immunoassay method was used for the measurement of cortisol (IMMULITE 2000 Cortisol, Siemens Healthcare Diagnostics Products Ltd. United Kingdom). All analyses were performed by Synlab Estonia.

## 5. Power calculation

The primary variable of the study was the level of 25(OH)D in the serum. In the absence of available pilot data, a pragmatic decision was taken in the power analysis; a difference of 20 nmol/l (i.e. less than the increments of 25 nmol/l in the study by Funderburk et al. (18)) between the intervention and the control group was considered to be the meaningful detectable difference. For example, if the SD would be 25 nmol/l, then 26 participants would be needed in each group to reach a power of 80%. Correspondingly, if the difference in hand grip strength would be 6 kilograms between the study groups, and the SD 8 kilograms, then 29 participants would be needed in each group to reach a power of 80%. Initially, 65 participants were recruited to the study to allow for dropouts.

## 6. Statistical analysis

The blood serum values and the hand grip strength in the study groups were described by medians, ranges, means, and standard deviations (SD). Differences in mean values of the variables between the groups were evaluated using an ANOVA test followed by Scheffe’s post hoc test. The within-group comparisons over time were performed using a Repeated Measures ANOVA followed by a Scheffe’s posthoc test. At all follow-up occasions, the blood serum value of 25(OH)D was dichotomously classified as < 25 nmol/l (critically low) or not and analyzed between the groups using Fisher’s exact test. For the primary variable, the 25(OH)D, the delta values at the follow-ups in relation to baseline values were calculated and reported as means and SDs. Statistical significance was set at p < 0.05.

## 7. Results

The mean baseline values of 25(OH)D in October 2016 were 49.8 nmol/l in the control group and 47.2 nmol/l in the intervention group (p = 0.63). At all subsequent time points, the control group had a significantly lower value of 25(OH)D (p < 0.001) compared to the intervention group. This is further emphasized because the delta value between the baseline and all follow-ups is significantly better in the intervention group (p < 0.001) (Table 2).

**Table 2.** Vitamin D 25(OH)D, PTH, testosterone, cortisol, calcium and ionized calcium serum level results.

	Baseline (October 2016)			Follow-up I (December 2016)			Follow-up II (March 2017)			Follow-up III (April 2017)		
**Blood serum tests** **(normal values)**	**Intervention** **group**	**Placebo** **group**	**p-value**	**Intervention** **group**	**Placebo** **group**	**p-value**	**Intervention** **group**	**Placebo** **group**	**p-value**	**Intervention** **group**	**Placebo** **group**	**p-value**
**Vitamin D 25(OH)D** **(75> nmol/l)** **Median (range)** **Mean (SD)**	47.8 (19.3-93.9) 47.2 (18.6)	50.0 (22.3 - 88.7) 49.8 (20.0)	n.s. (0.63)	62.5 (19.1-91.0) 59.8 (15.9)	36.8 (15.9-66.9) 36.4 (14.2)	<0.001	53.0 (12.1-88.4) 50.2 (19.8)	20.2 (11.1-37.3) 21.9 (7.7)	<0.001	52.2 (18.6-89.1) 53.6 (18.3)	28.9 (16.9-49.9) 29.9 (9.3)	<0.001
**Vitamin D 25(OH)D** **Delta value compared to baseline (nmol/l)** **Mean (SD)**	NA	NA		12.5 (16.5)	-13.4 (9.8)		2.9 (15.2)	-27.9 (13.4)		6.3 (20.2)	-19.9 (12.7)	
**Parathyroid hormon** **(1.48-7.83 pmol/l)** **Median (range)** **Mean (SD)**	4.5 (1.7-10.1) 4.8 (2.2)	4.4 (0.9-9.9) 4.5 (2.4)	n.s. (0.60)	3.3 (0.8-7.4) 3.6 (1.7)	3.9 (0.7-12.9) 4.3 (2.4)	n.s (0.20)	4.3 (1.2-6.0) 3.9 (1.6)	4.3 (1.8-20.1) 5.0 (3.6)	n.s. (0.14)	4.5 (1.8-8.2) 4.7 (1.8)	4.3 (2.2-8.8) 4.9 (2.1)	n.s. (0.75)
**Testosterone** **(8.4-28.7 nmol/l)** **Median (range)** **Mean (SD)**	15.4 (10.8-29.6) 17.4 (5.3)	16.2 (6.1-31.0) 17.4 (5.7)	n.s. (0.99)	20.0 (5.2-26.6) 18.7 (5.3)	18.9 (11.7-30.0) 19.3 (4.0)	n.s (0.65)	19.1 (8.3-26.0) 18.5 (4.2)	21.0 (5.7-28.4) 20.5 (4.8)	n.s. (0.12)	20.5 (10.1-32.5) 21.3 (5.9)	20.1 (13.0-28.4) 20.3 (4.1)	n.s. (0.48)
**Cortisol** **(138-690 nmol/l)** **Median (range)** **Mean (SD)**	527.0 (400-615) 517.3 (62.7)	506.5 (348-670) 513.7 (69.8)	n.s. (0.85)	538.0 (450-756) 554.0 (70.1)	561.5 (292-676) 536.1 (91.6)	n.s (0.43)	452.0 (251-629) 457.7 (105.1)	452.5 (114-577) 438.8 (94.4)	n.s. (0.50)	488.0 (265-668) 482.4 (85.8)	473.5 (287-599) 470.3 (76.6)	n.s. (0.59)
**Calcium** **(2.15-2.6 mmol/l)** **Median (range)** **Mean (SD)**	2.37 (2.28-2.50) 2.39 (0.07)	2.38 (2.23-2.53) 2.37 (0.07)	n.s. (0.30)	2.28 (2.18-2.36) 2.28 (0.05)	2.25 (2.13-2.43) 2.26 (0.06)	n.s (0.22)	2.36 (2.29-2.48) 2.36 (0.05)	2.36 (2.24-2.46) 2.35 (0.06)	n.s .(0.61)	2.29 (2.11-2.42) 2.28 (0.08)	2.26 (2.12-2.34) 2.24 (0.05)	0.05
**Calcium ionized** **(1.12-1.32 mmol/l)** **Median (range)** **Mean (SD)**	1.23 (1.15-1.31) 1.23 (0.04)	1.22 (1.15-1.28) 1.21 (0.03)	n.s. (0.23)	1.25 (1.20-1.32) 1.25 (0.03)	1.24 (1.17-1.32) 1.25 (0.04)	n.s (0.58)	1.23 (1.08-1.33) 1.22 (0.04)	1.23 (1.18-1.28) 1.23 (0.03)	n.s .(0.58)	1.23 (1.18-1.29) 1.24 (0.03)	1.23 (1.15-1.29) 1.23 (0.03)	n.s. (0.37)

Abbreviations: SD, standard deviation; n.s., non-significant

**Figure 2.**
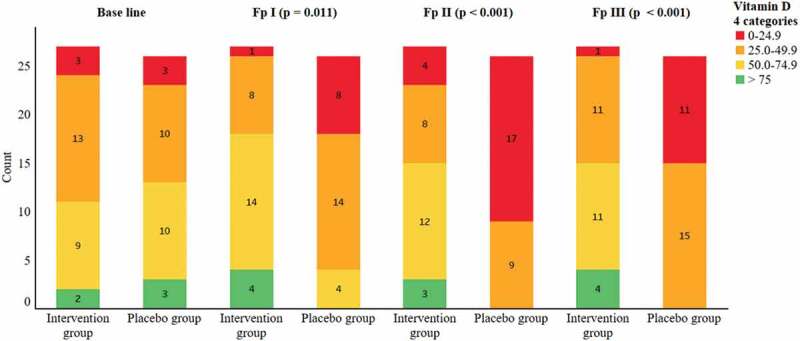
Distribution of vitamin D serum 25(OH)D levels in the intervention and control groups over the study period according to categories based on the Endocrine Society [1] and the study by Funderburk et al. [18]. Significantly more critically low values were found in the control group at all follow-up occasions (p = 0.011 follow-up I and p < 0.001 follow-up II and follow-up III).

In the intervention group, no significant decrease over time was seen. The average seasonal values of 25(OH)D for both groups are presented in Table 2. Seasonal within-group variation in 25(OH)D levels is presented in Figure 2. At baseline, there were three conscripts in each group with critically low 25(OH)D (<25 nmol/l) values. At all subsequent follow-up occasions, there were significantly more conscripts with critically low 25(OH)D values in the control group (p = 0.011 follow-up I and p < 0.001 follow-up II and follow-up III), (Figure 2). There were no conscripts with values higher than 50 nmol/l in the control group in April 2017.

No significant differences in Ca, Ca-i, PTH, testosterone, and cortisol levels were revealed between the study groups at any time point (Table 2). One participant in the control group had abnormally high PTH values throughout the study period, with serum 25(OH)D values lower than 25 nmol/l at all time points. No significant differences at any time points were revealed in the hand grip strength tests of either hand between the study groups (Table 3).

**Table 3.** Hand grip strength results for both hands during the study period.

	Baseline (October 2016)			Follow-up I (December 2016)			Follow-up II (March 2017)			Follow-up III (April 2017)		
	Intervention group	Placebo group	p-value	Intervention group	Placebo group	p-value	Intervention group	Placebo group	p-value	Intervention group	Placebo group	p-value
Hand grip right Median (range) Mean (SD)	48.0 (29–72) 48.0 (9.8)	50 (28–72) 49.1 (10.2)	n.s (0.70)	50.0 (30–65) 50.1 (7.6)	50.0 (30–70) 50.8 (8.5)	n.s. (0.74)	52.0 (30–80) 52.6 (9.6)	53.0 (30–70) 52.5 (10.0)	n.s. (0.97)	51.0 (28–84) 51.0 (9.0)	50.5 (34–68) 51.8 (9.0)	n.s. (0.78)
Hand grip left Median (range) Mean (SD)	46.0 (32–66) 45.9 (8.3)	50.0 (29–68) 47.8 (9.3)	n.s. (0.45)	48.0 (32–60) 47.9 (6.7)	49.0 (32–70) 49.5 (9.3)	n.s. (0.48)	50 (38–68) 50.8 (7.3)	52.0 (32–70) 50.8 (8.9)	n.s. (0.99)	49.0 (38–75) 49.9 (9.2)	50.0 (32–66) 50.7 (8.9)	n.s. (0.76)

Abbreviations: SD, standard deviation; n.s., nonsignificant

There were no reported side effects of the supplementation with vitamin D during the study period. No major injuries occurred in either study group.

## 8. Discussion

The main finding of the present study is that 7-month vitamin D3 supplementation results in fewer conscripts in the Estonian Army with critically low serum vitamin D (25(OH)D) levels during the winter season; however, this supplementation did not appear to affect the hand grip strength at any time point during the study period.

Despite no observed significant effect on hand grip strength in this study, the prevention of critically low vitamin D (25(OH)D) levels with vitamin D supplementation is noteworthy, particularly given the relationship of vitamin D deficiency with several other health and fitness parameters, such as bone metabolism, muscle function, metabolic disorders, and cardiovascular health [1,2,5].

Moreover, the decrease in the levels of 25(OH)D in the placebo group of this study reveals how crucial vitamin D supplementation is at Nordic latitudes, because the majority (65%) of the subjects in the placebo group presented critically low levels in March 2017, and none of them reached 50 nmol/l in April.

In 2016, the European Food Safety Authority (EFSA) panel considered that a serum 25(OH)D concentration of 50 nmol/l is a suitable target value for all population groups; this should be possible to achieve with a dietary intake of 15 µg/day (equal to 600 IU) [40].

The present study shows that during the winter season, the regular diet administered by the Estonian Army to conscripts does not provide enough vitamin D to achieve the 50 nmol/l level suggested by the EFSA panel. The exact amount of vitamin D in the regular Estonian Army diet is not known. However, the diet of the conscripts is based on guidelines from professional dieticians who have calculated the daily requirements of energy, minerals, and vitamins (Personal communication. ‘Regulation of dietary norms for soldiers’, regulation no. 240 of the Commander of the Defense Forces of the Estonian Army, September 13^th^, 2013).

Vitamin D has a stimulating effect on muscle protein synthesis [9,41] and has been shown to play a crucial biomolecular role in skeletal muscle activation and, thus, muscle function [3,42]. Therefore, vitamin D deficiency might have a detrimental effect during physically demanding situations, such as military service.

Testosterone and cortisol play crucial roles in muscle fitness, physical performance, and general health [13]. Vitamin D is linked to testosterone production in the human body, and supplementation of vitamin D has shown a positive effect on testosterone levels [43,44]. It is also known that increased levels of testosterone can improve physical performance [45,46]. Cortisol, commonly called the stress hormone, is one of the glucocorticoids, and it plays an important role in regulating muscle function, energy homeostasis, metabolism, and adaptation for physical exercises [47]. However, increased levels of cortisol can decrease physical performance ability [48]. Interestingly, no significant differences between the study groups were revealed in either testosterone or cortisol levels at any time point in the present study. This might be due to the fact that either the study group was small or summertime monitoring was not performed. Lombardi et al. [49] found higher levels of cortisol during wintertime and higher levels of testosterone during summertime in a group of 167 elite football players, which also correlated with vitamin D seasonal differences. Mielgo-Ayuso et al. [45] found that short-term (eight weeks) supplementation with 3000 IU vitamin D in 36 elite male rowers did not increase muscle recovery; however, they did find that serum 25(OH)D levels were predictors of both anabolic and catabolic hormone levels. On the other hand, a recent study of 50 young male ice hockey players found no statistically significant associations between 25(OH)D and testosterone and cortisol concentrations in a single blood test in the month of October [50]. In line with this, Worzosek et al. [51], in a study of 55 male athletes who received 12 weeks of 2000 IU vitamin D supplementation, found no effect on the levels of testosterone, estradiol, or cortisol, which is similar to the findings of the present study.

Normative mean values for hand grip strength across people in the same age group as those in the present study vary globally [52]: 42.2 kg in Korea [53], 41.5 kg in Great Britain [54], and 47.0 kg in the USA [55]. Normative values in this age group in the Estonian male population are unknown, which of course is a general weakness. In the present study, the mean values of hand grip strength ranged from 45.9 kg to 52.6 kg, which is in line with other studies in the literature. Due to the high risk of critical wintertime deficiency of 25(OH)D in the Estonian young male population [19] and evidence in the literature that vitamin D plays a role in hand grip strength [56,57], the authors still expected a positive effect of supplementation. Despite low 25(OH)D levels in the springtime in the control group, there were no differences in the hand grip strength of either hand between the study groups.

There is still lack of knowledge in terms of the role of vitamin D in upper limb muscle strength in the general population. The studies by Haslam et al. [58] and Wang et al. [59] showed that vitamin D deficiency is related to loss of hand grip strength in the older population, and there is evidence that vitamin D also plays a key role in younger age groups for upper limb muscle strength [6,56,57,60].

For people involved in physically demanding activities, such as army service, there is probably a higher demand for vitamin D supplementation than that shown in previous studies. This is supported by multiple studies showing vitamin D deficiency in army recruits [26,27,30,61]. Willis et al. [62] found that a surprisingly high proportion of athletes have a vitamin D deficiency; 77% of German gymnasts had 25(OH)D levels below 35 ng/ml (87.5 nmol/L), and 37% had a critical deficiency value of < 10 ng/ml (< 25 nmol/L) [62].In a study by Allison et al. [63], it was reported that severely vitamin-D-deficient athletes have smaller hearts than athletes with moderate vitamin D deficiency or insufficiency, but a similar effect was not observed in healthy, nonathletic controls.

Hughes et al. [64] showed in a study in the US Army that under military training, bone resorption is increased and bone formation slowed down. In their study, bone metabolism was normalized 2 to 6 weeks after cessation of training [64]. According to this prospective study of 3787 soldiers participating in military operations over 15 months, 19% needed an orthopedic consultation, and for 4%, orthopedic surgery was indicated [65]. Vitamin D plays a very important role in bone metabolism, and it has been shown that vitamin D and calcium supplementation can prevent stress fractures during military service. A large controlled, randomized, double-blinded study of 5201 female navy recruits in the US Army demonstrated that calcium and vitamin D supplementation resulted in a 20% lower incidence of stress fractures than in a control group [66].

Vitamin D deficiency also plays a crucial role in the human immune system at the cellular level [67]. Laaksi et al. [27] detected an association between low 25(OH)D levels and occurrence of respiratory infections in 800 Finnish conscripts. In a randomized, double-blinded study, performed by the same authors on 164 conscripts over a 6-month period during the winter season, a 400 IU vitamin D supplementation was compared to a placebo. This study revealed that the frequency of acute respiratory infections was significantly lower in the supplementation group [25]. Similar findings were detected in college athletes in the USA in a prospective follow-up study monitoring vitamin D status [4].

The recommendations of the ES [1] are similar to the EFSA 2016 recommendations [40] for young, healthy males under normal civil conditions. However, the daily Recommended Dietary Allowance (RDA) of the ES for risk groups is 1500–2000 IU [40]. Military service can be considered a risk factor for vitamin D deficiency [9,63,64]. Despite the fact that a double dose based on ES or EFSA recommendations was used in the present study, it was still insufficient to improve the 25(OH)D serum levels in Estonian Army conscripts during an intensive winter season training period. It has been debated that under physically demanding situations such as athletic performance, the usual amounts of vitamin D supplementation may be insufficient. It has been speculated that the supplementation amounts should instead be 1000–2000 IU of vitamin D per day [62].

In a cross-sectional study based on all age groups of the Estonian population, Kull et al. [19] found low values of 25(OH)D, similar to those in the control group of the present study.

The northern latitude of Estonia, as well as the high physical demand of military service, apparently exposes conscripts to an increased risk of vitamin D deficiency. Thus, higher doses may be needed in order to prevent vitamin D deficiency in this group.

It would be interesting to follow young individuals in the northern latitudes over a longer period of time to evaluate the effect of vitamin D supplementation on their performance and well-being.

The role of vitamin D in physical performance needs more studies incorporating other physical performance tests and other supplementation protocols using a higher daily dose of vitamin D. Future studies should not only be performed on conscripts, but on the general population as well. Higher vitamin D supplementation doses can now be used, since up to 4000 IU per day is now authorized by the Estonian State Agency of Medicines.

In terms of PTH levels, during the present study, there were no significant changes detected over time. This is in concordance with earlier reports that PTH decreases during the summer–autumn season [68], which was unfortunately outside the follow-up period of the present study.

It is known that excessively high doses of Vitamin D or blood serum levels of 25(OH)D together with low calcium intake can increase the calcium level outside the normal range, causing an increase in bone resorption and a decrease in bone mineralization [69]. There is also a risk of hypercalcemia causing general gastrointestinal problems such as constipation and hypercalciuria with renal calculi. Myocardial infarction, stroke, vascular disease, and even death have been reported after intake of calcium supplements [70]. In the present study, the calcium levels were normal during the study period, indicating that a dose of at least 1200 IU can safely be used.

The strengths of this study lie in its randomized and blinded design, the homogenous test group, and the follow-up period over the whole winter season with four physical performance test occasions. Until now, there have been only short-term (8–12 weeks of follow-up), randomized studies performed on soldiers under physically demanding activities to examine the effect of vitamin D supplementation [4,30,58,71]. The present study examines the effect of supplementation during the whole winter season. The study population was well standardized in terms of conditions, such as the season of the year, age, sex, daily food consumption, state of dress, and physical activity. This provides an ideal opportunity to study the effect of vitamin D supplementation on serum levels of 25(OH)D and physical performance in the form of the hand grip test.

The limitations of the study include the small groups, relatively short wintertime follow-up of 7 months, the use of only one supplementation dosage, the use of only one physical performance test, the exclusion of female subjects, the missing of lean body mass measurements, and registration of general health problems and acute respiratory infections. Due to participant dropouts, the study is underpowered for the secondary outcome variable, the hand grip test. Also, the hand grip strength test might be a less sensitive test in the young population; lots of participants score highly, and an improvement might be hard to detect. Due to the expected seasonal variation of serum levels of 25(OH)D, the authors decided not to use the intention-to-treat principle and only analyze those participants who attended all follow-up occasions, which can also be considered a weakness. Furthermore, many conscripts chose not to participate. However, it can be assumed that there are either no or only small differences between conscripts because of the homogeneity of soldiers in the Estonian Army. Finally, the generalizability of the study is limited to military personnel in relatively high northern or southern latitudes.

## 9. Conclusion

Long-term vitamin D3 supplementation results in fewer conscripts in the Estonian Army with critically low serum vitamin D (25(OH)D) levels during the winter season. However, this did not influence their physical performance in the form of the hand grip strength test.

## List of abbreviations

BMI: body mass index
EFSA: European Food Safety Authority
ES: Endocrine Society
Ca: calcium
Ca-I: ionized calcium; (Ca-i)
SD: standard deviation
n.s: non-significant
PTH: parathyroid hormone
RDA: Recommended Dietary Allowance.

## Data Availability

The data sets generated and/or analyzed during the current study are available in the datadoi.ee University of Tartu Library repository, https://datadoi.ee/handle/33/342, http://dx.doi.org/10.23673/re-284
